# Ependymal cells of the mouse brain express urate transporter 1 (URAT1)

**DOI:** 10.1186/2045-8118-10-31

**Published:** 2013-10-24

**Authors:** Naoko H Tomioka, Makiko Nakamura, Masaru Doshi, Yoshiharu Deguchi, Kimiyoshi Ichida, Takayuki Morisaki, Makoto Hosoyamada

**Affiliations:** 1 Department of Human Physiology and Pathology, Faculty of Pharma-Sciences, Teikyo University, 2-11-1 Kaga, Itabashi-ku, Tokyo 173-8605, Japan; 2 Department of Pathophysiology, Tokyo University of Pharmacy and Life Sciences, 1432-1 Horinouchi, Hachiouji, Tokyo 192-0392, Japan; 3 Department of Drug Disposition and Pharmacokinetics, Faculty of Pharma-Sciences, Teikyo University, 2-11-1 Kaga, Itabashi-ku, Tokyo 173-8605, Japan; 4 Department of Bioscience and Genetics, National Cerebral and Cardiovascular Center Research Institute, 5-7-1 Fujishirodai, Suita, Osaka 565-8565, Japan

**Keywords:** Uric acid, URAT1, Ependymal cells, Cilia

## Abstract

**Background:**

Elevated uric acid (UA) is commonly associated with gout and it is also a known cardiovascular disease risk factor. In contrast to such deleterious effects, UA possesses neuroprotective properties in the brain and elucidating the molecular mechanisms involved may have significant value regarding the therapeutic treatment of neurodegenerative disease. However, it is not yet fully established how UA levels are regulated in the brain. In this study, we investigated the distribution of mouse urate transporter 1 (URAT1) in the brain. URAT1 is a major reabsorptive urate transporter predominantly found in the kidney.

**Methods:**

Immunohistochemistry of wild type and URAT1 knockout mouse brain using paraffin or frozen sections and a rabbit polyclonal anti-mouse URAT1 antibody were employed.

**Results:**

Antibody specificity was confirmed by the lack of immunostaining in brain tissue from URAT1 knockout mice. URAT1 was distributed throughout the ventricular walls of the lateral ventricle, dorsal third ventricle, ventral third ventricle, aqueduct, and fourth ventricle, but not in the non-ciliated tanycytes in the lower part of the ventral third ventricle. URAT1 was localized to the apical membrane, including the cilia, of ependymal cells lining the wall of the ventricles that separates cerebrospinal fluid (CSF) and brain tissue.

**Conclusion:**

In this study, we report that URAT1 is expressed on cilia and the apical surface of ventricular ependymal cells. This is the first report to demonstrate expression of the urate transporter in ventricular ependymal cells and thus raises the possibility of a novel urate transport system involving CSF.

## Background

Uric acid (UA) is the end product of purine metabolism in human and hyperuricemia is a known risk factor for gout, hypertension, renal disease, and metabolic syndrome [1]. While high levels of UA are considered as a pathogenic factor, UA also possesses antioxidant properties and epidemiological studies have shown that reduced concentrations of UA are linked to neurodegenerative disorders such as multiple sclerosis, Parkinson’s disease (PD), Alzheimer’s disease and optic neuritis [2], supporting the potential neuroprotective effect of UA. In addition to human epidemiological studies, the neuroprotective effect of UA has also been identified in cellular and animal models for PD [3-5].

Physiologically, UA concentrations are an order of magnitude lower in cerebral tissue compared to blood UA levels [6]. Interestingly, studies have reported that CSF and blood UA levels are correlated [5,7]. Furthermore, increased plasma and striatum levels of UA have been detected in rats given intraperitoneal injections of UA [4]. These reports suggest that UA in the brain, and CSF, might be dependent upon the level of UA in the blood, while UA could also be synthesized locally in the brain [5]. However, due to a significant lack of information regarding the key molecules involved, the origin of brain UA, or how UA homeostasis is maintained in brain parenchyma and CSF, remains largely unknown.

Multiple urate transporters in the kidney are involved in the regulation of serum UA levels (SUA). Renal urate reabsorption is mainly mediated by urate transporter 1 (URAT1) on the apical side of the renal proximal tubular cells, and the voltage-driven urate transporter (URATv1/Glut9) on the basolateral side [8,9]. In addition, the ATP-binding cassette transporter, sub-family G, member 2 (ABCG2), has been identified as another transporter that regulates SUA [10-12]. Intriguingly, while the presence of these transporters in the brain has been demonstrated in several studies [13-16], detailed spatial information of the specific localization pattern of these vital transporters is generally lacking.

In the present study, we investigated the distribution of URAT1 in mouse brain by immunohistochemical analysis using an anti-URAT1 antibody [17], the specificity of which was confirmed using a URAT1 knockout mouse.

## Methods

### Animals

Male C57BL/6 J mice (Sankyo Laboratories, Tokyo, Japan) and homozygous URAT1 knockout mice [17] were maintained under a 14:10 light cycle with free access to food and water. For this study, 8–10 mice were used in each group. All animal experiments were carried out in accordance with the guidelines for animal experimentation in Teikyo University and the project was approved by the local committee.

### Immunohistochemistry of URAT1 in mouse brain

Nine-week-old C57BL/6 J male mice and URAT1 knockout male mice were anesthetized by pentobarbital injection (50 mg/kg, i.p.) and perfused intra-cardially with 4% paraformaldehyde in HEPES buffer (30 mM HEPES, 100 mM NaCl, 2 mM CaCl_2_, pH 7.5). Brains were removed and post-fixed overnight (for paraffin embedding) or 3 h (for cryoprotection) at 4°C in the same fixative. Brains were then cut coronally or sagittally and prepared for paraffin embedding or cryoprotection. For paraffin embedding, tissues were dehydrated in a graded series of alcohols, cleared with Hemo-De (a Xylene substitutive, FALMA, Tokyo, Japan), embedded in Paraplast (Sigma, St. Louis, MO, USA), and sectioned at 3 μm. After deparaffinization and rehydration, endogenous peroxidase activity was blocked by 0.03% H_2_O_2_ methanol solution for 10 min. Sections were then incubated for 1 h at room temperature in blocking solution containing 2% bovine serum albumin (Jackson ImmunoResearch, West Grove, PA, USA) in 0.01 M PBS with 0.02% Tween-20, followed by incubation with primary antibodies diluted in blocking solution at 4°C overnight (anti-URAT1 antibody, 1:5000). Immunostaining was visualized with EnVision™ Systems and DAB+, Liquid (Dako Japan, Tokyo Japan). To prepare cryostat sections (12 μm), post-fixed brains were cryoprotected in 15% sucrose (wt/vol) in PBS for 48 h at 4°C, embedded in Tissue-Tek OCT compound (Sakura Finetek Japan, Tokyo, Japan), and frozen on dry ice. Cryostat sections were fixed in methanol (−20°C, 30 min) and acetone (4°C, 10 min), incubated for 1 h at room temperature in blocking solution containing 5% normal goat serum (Cedarlane, Ontario, Canada) in 0.01 M PBS with 0.1% Triton X-100, followed by incubation with primary antibodies diluted in blocking solution at 4°C overnight. The following antibodies were used for double staining: anti-URAT1, 1:1000; anti-acetylated-α-tubulin (6-11B-1, IgG2b, Sigma), 1:500. Sections were then incubated with Alexa 488- or Alexa 594-conjugated secondary antibodies (Life Technologies, Tokyo, Japan) for 1 h at room temperature and mounted with Vectashield containing DAPI (Vector Laboratories, Burlingame, CA, USA).

### Image acquisition

DAB-stained sections were scanned with a NanoZoomer 2.0-HT slide scanner (Hamamatsu Photonics, Hamamatsu, Japan). For immunofluorescence experiments, single-plane images were captured using a Nikon A1 confocal microscope (Nikon, Tokyo, Japan) with identical settings.

## Results

### Distribution of URAT1 in mouse brain

**Figure 1:**
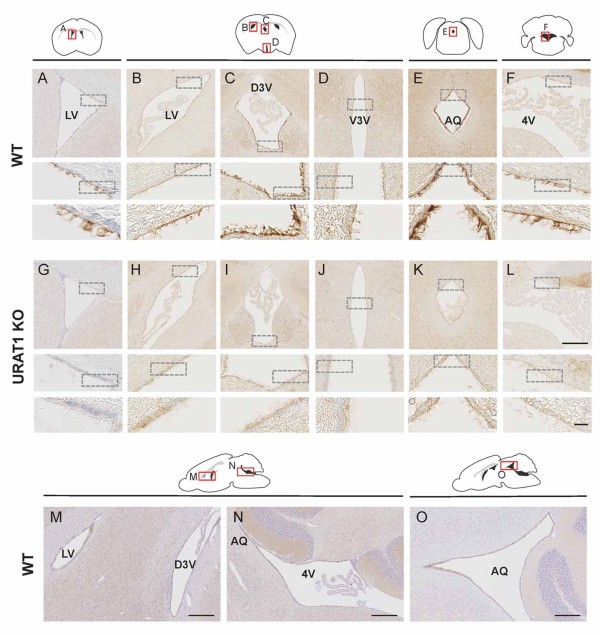
**Distribution of URAT1 in mouse brain.** Paraffin sections from nine-week-old WT (**A–F**, coronal section; **M–O**, sagittal section) and URAT1 KO (**G–L**, coronal section) mice were stained with a polyclonal antibody to URAT1 (anti-URAT1 antibody) and counterstained with hematoxylin. Red squares in the diagrams indicate the region of the images while the lower panels show magnified images around the ependymal cells (represented as dotted squares in the upper panels). Images **(B–D)**, **(H–J)** or **(M–N)** were obtained from the same sections, respectively. Scale bars, 200 μm and 10 μm in top and bottom row of **(L)**, respectively; 200 μm in **(M–O)**. LV, lateral ventricle; V3V, ventral third ventricle; D3V, dorsal third ventricle, AQ, aqueduct; 4V, fourth ventricle.

To investigate the distribution of URAT1 in the mouse brain, we performed immunohistochemistry upon adult mouse brain paraffin coronal sections by using a rabbit polyclonal anti-mouse URAT1 antibody [17]. Strong URAT1 immunoreactivity was detected in ependymal cells lining the lateral ventricle (Figure 1A and B), dorsal third ventricle (Figure 1C), ventral third ventricle (Figure 1D), aqueduct (Figure 1E) and fourth ventricle (Figure 1F). These patterns of immunoreactivity were not observed in sections from the URAT1 knockout mice (Figure 1G–L). Weaker immunostaining in the brain parenchyma, and the choroid plexus of the lateral and dorsal third ventricles, appeared to be non-specific, since similar staining was observed in sections from the knockout mice (Figure 1).

Ependymal cells form an epithelial layer that lines the cerebral ventricles and function as a barrier between the cerebrospinal fluid and the brain parenchyma. Morphologically, these cells possess cilia and microvilli on their apical surfaces. At higher magnification, dense URAT1 positive signals were detected in cilia-like protrusions (Figure 1A–F, lower panels). URAT1 localization along the ventral third ventricle was restricted to the ependymal cells that line the upper part of the ventral third ventricle and could not be observed in the non-ciliated tanycytes, which are located in the lower part of the ventral third ventricle (Figure 1D).

URAT1-distribution in the ventricles was also examined in sagittal sections, showing continuous expression in the lateral ventricle (Figure 1M), dorsal third ventricle (Figure 1M), aqueduct (Figure 1N and O) and fourth ventricle (Figure 1N). These results suggest that URAT1 is expressed in ciliated-ependymal cells in the ventricular system of the mouse brain.

### Expression levels of URAT1 differ between ventricles

**Figure 2:**
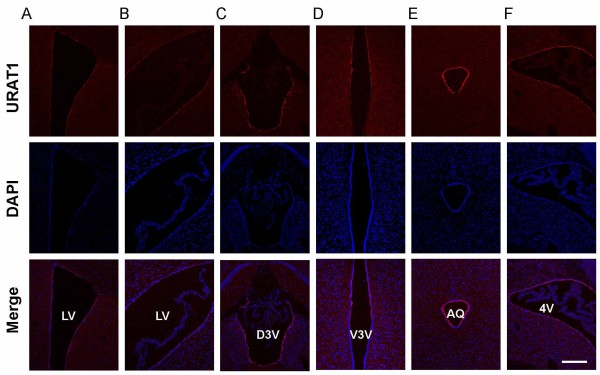
**Expression levels of URAT1 differ between ventricles. (A–F)** Frozen coronal sections from nine-week-old WT mice were stained with anti-URAT1 (red) and DAPI (blue) and imaged by confocal microscopy. Scale bar, 200 μm.

Interestingly, URAT1 immunoreactivity in the aqueduct was prominent compared to the other ventricular regions (Figure 1E,N,O). To confirm this point, we performed immunofluorescence staining of frozen sections using the same anti-URAT1 antibody. Similar to the results gleaned from paraffin section immunohistochemistry, intense immunoreactivity was distributed in the ependymal cells of multiple ventricles, but not at the choroid plexus or in the brain parenchyma (Figure 2A–F). In the ventral third ventricle, URAT1-positive signals were restricted to the upper part of the ventral third ventricle and not detected in tanycytes of the lower part of the ventral third ventricle (Figure 2D). Within the ventricular system, the strongest signal was observed in the aqueduct (Figure 2E), indicating that expression levels of URAT1 differ between ventricles.

### URAT1 localizes to the apical surface, including the cilia of ependymal cells

**Figure 3:**
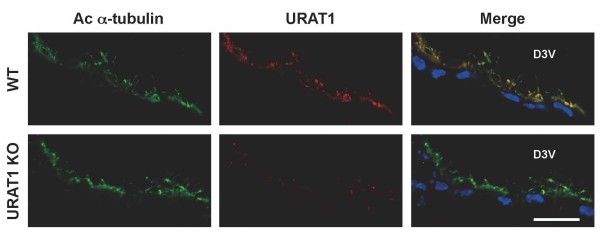
**URAT1 localizes at the apical surface of ependymal cells.** Frozen coronal sections from nine-week-old WT and URAT1 KO mice were stained with anti-acetylated-α-tubulin antibody (green), anti-URAT1 antibody (red) and DAPI (blue). The cilia of the ependymal cells lining the D3V were visualized by anti-acetylated-α-tubulin antibody as a cilia marker. Scale bar, 20 μm.

Finally, we performed double-immunostaining using an anti-URAT1 antibody and anti-acetylated-α-tubulin antibody as a marker for cilia (Figure 3). At high magnification, URAT1 localized to the apical but not to the basolateral membrane of the ependymal cells, which largely co-exist with acetylated-α-tubulin. The absence of URAT1 in the cilia did not affect the localization of acetylated-α-tubulin, indicating that cilia morphology is intact in the knockout mouse. Reminiscent of the URAT1 expression at the apical brush-border membrane of the renal tubular cells, URAT1 localized to the apical membrane including the cilia of ependymal cells.

## Discussion

In the present study, we suggest that the urate transporter URAT1 is involved in UA transport in the ventricular ependymal cells of the mouse brain. Using immunohistochemistry, we showed that URAT1 is expressed on cilia and the apical surface of ependymal cells surrounding the ventricles, and the absence of such staining was confirmed in URAT1 knockout mice. Although URAT1 expression in brain tissues such as brain capillaries, choroid plexus [14], cultured dopaminergic cell lines and astrocytes [18] has been demonstrated by immunoblot analyses, we could not observe comparable expression of URAT1 by immunohistochemical analyses in such regions as we did in ependymal cells. Weaker immunostaining in the brain parenchyma and the choroid plexus were observed in sections from both wild type and knockout mice. Therefore, the band identified by immunoblotting in earlier studies might represent a non-specific binding to a protein other than URAT1. Moreover, earlier studies failed to check whether ependymal cells may have contaminated the brain capillary, choroid plexus or astrocyte samples which were used for immunoblotting. Thus, the expression of URAT1 protein in brain capillaries, choroid plexus or astrocytes has not been verified completely. To our knowledge, this is the first report to demonstrate urate transporter localization in ventricular ependymal cells.

Ependymal cells are single-layered ciliated epithelial cells lining the ventricular surface of the central nervous system, and act as the brain-CSF interface. This site is responsible for the exchange of substances between CSF and extracellular fluid in the neuropile [19]. Since URAT1 transports UA via a mechanism involving the exchange of lactate [8], we propose a novel mechanism in which UA in the CSF is absorbed intracellularly by ependymal cells in the presence of an outwardly-directed lactate gradient. There are two conceivable possibilities for the formation of this lactate gradient. As in the renal tubular cells, a lactate gradient can be formed by the sodium-coupled monocarboxylate transporters SMCT1 and SMCT2, which collectively facilitate the reabsorption of UA via URAT1 [20]. However, the expression of SMCT1 and SMCT2 have only been demonstrated in neurons and Muller glial cells, respectively [21,22], and the expression and possible involvement of SMCTs in ependymal cells remains unknown. Alternatively, the difference in basal lactate concentration between CSF and ependymal cells may be important. Both human and rat data show that lactate concentration is higher in brain ECF (2–5 mM) compared to that in blood (1–2 mM) [23]. In humans, levels of CSF lactate range from 2.0 to 2.7 mM [24]. Since other lactate transporter proteins, referred to as MCT1, MCT2 and MCT4 (monocarboxylate transporter 1, 2 and 4), have been identified in ependymal cells, probably on the basolateral side [25-29], the lactate levels in ependymal cells may be equivalent to those of brain parenchyma, and thus likely to be higher than CSF lactate. Further analysis regarding the formation of a lactate gradient would be essential in understanding the UA transporting function of URAT1 in ependymal cells.

One outstanding question is how UA might function following incorporation into ependymal cells. One possibility is that intracellular UA may act as an antioxidant and maintain the redox state of ependymal cells. These cells have been shown to contain high levels of glutathione (GSH) [30], a major antioxidant in the CNS, suggesting a need for antioxidant mechanisms. Intriguingly, in the ventral third ventricle, we observed positive staining of URAT1 only in ciliated ependymal cells, but not in the tanycytes, which are non-ciliated cells lining the lower part of the ventral third ventricle. Intracellular uptake of UA may thus be important for the specific function of ciliated-ependymal cells such as ciliary beating, an essential mechanism for proper CSF flow [31].

Another possibility is that UA incorporated into the ependymal cells is further transported into the brain parenchyma. The fact that we detected strong URAT1 expression in ependymal cells may indicate a novel trans-cellular transport mechanism for UA across ependymal cells. This is reminiscent of the UA transport system in the proximal renal tubular cells, in which UA is reabsorbed by URAT1 in the apical membrane and effluxed by URATv1/Glut9 in the basolateral membrane. If urate efflux transporters exist on the basolateral side of ependymal cells, then it is possible that CSF-derived UA could pass through the brain parenchyma and be finally incorporated into neurons and/or astrocytes. One could speculate that UA transport may occur at the blood–brain barrier (BBB). However, the provision of UA from blood to brain parenchyma seems unlikely, since at this point, immunohistochemical data indicate that the urate efflux transporter ABCG2 is the sole UA transporter localized on the luminal side of brain capillaries [13]. Collectively, the URAT1-mediated transport of UA via ependymal cells represents a promising candidate for a key molecule involved in the regulation of UA homeostasis in the brain.

Based on epidemiological and clinical data showing that higher UA levels are associated with reduced risk and slower progression of PD, UA-elevating strategies have emerged as a potential therapy for PD [5]. Furthermore, attenuated neurodegeneration has been demonstrated in animal models of PD which showed increased levels of UA in response to the genetic manipulation of the UA-degrading enzyme, urate oxidase [3], or by UA injection [4], providing a convincing rationale for UA elevation. However, systemic elevation of UA is clearly pathogenic and the benefit-risk ratio of a therapeutic UA-elevating strategy remains uncertain. If the UA transport activity of URAT1 in ependymal cells is critical for the provision of UA to the brain, then the selective facilitation of URAT1 activity, and/or up-regulation of ependymal URAT1 expression in the brain, may be a beneficial strategy for developing UA-based therapy against neurodegenerative diseases.

## Conclusions

Our data suggests that the urate transporter URAT1 is expressed on cilia and the apical surface of ventricular ependymal cells in mouse brain. Ependymal URAT1 may thus be involved in a novel UA transport mechanism between CSF and brain parenchyma.

## Abbreviations

CSF: Cerebrospinal fluid; UA: Uric acid; PD: Parkinson’s disease; SUA: Serum UA levels.

## Competing interests

The authors declare that they have no competing interests.

## Authors’ contributions

NHT carried out immunohistochemical studies and drafted the manuscript. MN, KI, and TM carried out studies on URAT1 knockout mice. MD and YD designed the study. MH conceived the study, participated in its design and coordination, and helped draft the manuscript. All authors read and approved the final manuscript.

## References

[B1] Jin M Yang F Yang I Yin Y Luo JJ Wang H Yang XF Uric acid, hyperuricemia and vascular diseases Front Biosci 2012 10 656 669 10.2741/3950 PMC3247913 22201767

[B2] Kutzing MK Firestein BL Altered uric acid levels and disease states J Pharmacol Exp Ther 2008 10 1 7 17890445 10.1124/jpet.107.129031

[B3] Chen X Burdett TC Desjardins CA Logan R Cipriani S Xu Y Schwarzschild MA Disrupted and transgenic urate oxidase alter urate and dopaminergic neurodegeneration Proc Natl Acad Sci USA 2013 10 300 305 10.1073/pnas.1217296110 23248282 PMC3538212

[B4] Gong L Zhang QL Zhang N Hua WY Huang YX Di PW Huang T Xu XS Liu CF Hu LF Luo WF Neuroprotection by urate on 6-OHDA-lesioned rat model of Parkinson’s disease: linking to Akt/GSK3beta signaling pathway J Neurochem 2012 10 876 885 10.1111/jnc.12038 23094836

[B5] Chen X Wu G Schwarzschild MA Urate in Parkinson’s disease: more than a biomarker? Curr Neurol Neurosci Rep 2012 10 367 375 10.1007/s11910-012-0282-7 22580741

[B6] O’Neill RD Lowry JP On the significance of brain extracellular uric acid detected with in-vivo monitoring techniques: a review Behav Brain Res 1995 10 33 49 10.1016/0166-4328(95)00035-6 8747173

[B7] Bowman GL Shannon J Frei B Kaye JA Quinn JF Uric acid as a CNS antioxidant J Alzheimers Dis 2010 10 1331 1336 20061611 10.3233/JAD-2010-1330 PMC2859185

[B8] Enomoto A Kimura H Chairoungdua A Shigeta Y Jutabha P Cha SH Hosoyamada M Takeda M Sekine T Igarashi T Matsuo H Kikuchi Y Oda T Ichida K Hosoya T Shimokata K Niwa T Kanai Y Endou H Molecular identification of a renal urate anion exchanger that regulates blood urate levels Nature 2002 10 447 452 12024214 10.1038/nature742

[B9] Anzai N Ichida K Jutabha P Kimura T Babu E Jin CJ Srivastava S Kitamura K Hisatome I Endou H Sakurai H Plasma urate level is directly regulated by a voltage-driven urate efflux transporter URATv1 (SLC2A9) in humans J Biol Chem 2008 10 26834 26838 10.1074/jbc.C800156200 18701466

[B10] Dehghan A Kottgen A Yang Q Hwang SJ Kao WL Rivadeneira F Boerwinkle E Levy D Hofman A Astor BC Benjamin EJ Van Duijn CM Witteman JC Coresh J Fox CS Association of three genetic loci with uric acid concentration and risk of gout: a genome-wide association study Lancet 2008 10 1953 1961 10.1016/S0140-6736(08)61343-4 18834626 PMC2803340

[B11] Kolz M Johnson T Sanna S Teumer A Vitart V Perola M Mangino M Albrecht E Wallace C Farrall M Johansson A Nyholt DR Aulchenko Y Beckmann JS Bergmann S Bochud M Brown M Campbell H Consortium E Connell J Dominiczak A Homuth G Lamina C McCarthy MI Consortium E Meitinger T Mooser V Munroe P Nauck M Peden J Meta-analysis of 28,141 individuals identifies common variants within five new loci that influence uric acid concentrations PLoS Genet 2009 10 e1000504 10.1371/journal.pgen.1000504 19503597 PMC2683940

[B12] Kamatani Y Matsuda K Okada Y Kubo M Hosono N Daigo Y Nakamura Y Kamatani N Genome-wide association study of hematological and biochemical traits in a Japanese population Nat Genet 2010 10 210 215 10.1038/ng.531 20139978

[B13] Cooray HC Blackmore CG Maskell L Barrand MA Localisation of breast cancer resistance protein in microvessel endothelium of human brain Neuroreport 2002 10 2059 2063 10.1097/00001756-200211150-00014 12438926

[B14] Imaoka T Kusuhara H Adachi-Akahane S Hasegawa M Morita N Endou H Sugiyama Y The renal-specific transporter mediates facilitative transport of organic anions at the brush border membrane of mouse renal tubules J Am Soc Nephrol 2004 10 2012 2022 10.1097/01.ASN.0000135049.20420.E5 15284287

[B15] Augustin R Carayannopoulos MO Dowd LO Phay JE Moley JF Moley KH Identification and characterization of human glucose transporter-like protein-9 (GLUT9): alternative splicing alters trafficking J Biol Chem 2004 10 16229 16236 10.1074/jbc.M312226200 14739288

[B16] Keembiyehetty C Augustin R Carayannopoulos MO Steer S Manolescu A Cheeseman CI Moley KH Mouse glucose transporter 9 splice variants are expressed in adult liver and kidney and are up-regulated in diabetes Mol Endocrinol 2006 10 686 697 16293642 10.1210/me.2005-0010

[B17] Hosoyamada M Takiue Y Morisaki H Cheng J Ikawa M Okabe M Morisaki T Ichida K Hosoya T Shibasaki T Establishment and analysis of SLC22A12 (URAT1) knockout mouse Nucleosides Nucleotides Nucleic Acids 2010 10 314 320 10.1080/15257771003738634 20544513

[B18] Cipriani S Desjardins CA Burdett TC Xu Y Xu K Schwarzschild MA Protection of dopaminergic cells by urate requires its accumulation in astrocytes J Neurochem 2012 10 172 181 10.1111/j.1471-4159.2012.07820.x 22671773 PMC3438313

[B19] Del Bigio MR Ependymal cells: biology and pathology Acta Neuropathol 2010 10 55 73 10.1007/s00401-009-0624-y 20024659

[B20] Anzai N Kanai Y Endou H New insights into renal transport of urate Curr Opin Rheumatol 2007 10 151 157 10.1097/BOR.0b013e328032781a 17278930

[B21] Martin PM Dun Y Mysona B Ananth S Roon P Smith SB Ganapathy V Expression of the sodium-coupled monocarboxylate transporters SMCT1 (SLC5A8) and SMCT2 (SLC5A12) in retina Invest Ophthalmol Vis Sci 2007 10 3356 3363 10.1167/iovs.06-0888 17591909

[B22] Martin PM Gopal E Ananth S Zhuang L Itagaki S Prasad BM Smith SB Prasad PD Ganapathy V Identity of SMCT1 (SLC5A8) as a neuron-specific Na + −coupled transporter for active uptake of L-lactate and ketone bodies in the brain J Neurochem 2006 10 279 288 10.1111/j.1471-4159.2006.03878.x 16805814

[B23] Abi-Saab WM Maggs DG Jones T Jacob R Srihari V Thompson J Kerr D Leone P Krystal JH Spencer DD During MJ Sherwin RS Striking differences in glucose and lactate levels between brain extracellular fluid and plasma in conscious human subjects: effects of hyperglycemia and hypoglycemia J Cereb Blood Flow Metab 2002 10 271 279 11891432 10.1097/00004647-200203000-00004

[B24] Leen WG Willemsen MA Wevers RA Verbeek MM Cerebrospinal fluid glucose and lactate: age-specific reference values and implications for clinical practice PLoS One 2012 10 e42745 10.1371/journal.pone.0042745 22880096 PMC3412827

[B25] Gerhart DZ Enerson BE Zhdankina OY Leino RL Drewes LR Expression of monocarboxylate transporter MCT1 by brain endothelium and glia in adult and suckling rats Am J Physiol 1997 10 E207 E213 9252498 10.1152/ajpendo.1997.273.1.E207

[B26] Gerhart DZ Enerson BE Zhdankina OY Leino RL Drewes LR Expression of the monocarboxylate transporter MCT2 by rat brain glia Glia 1998 10 272 281 10.1002/(SICI)1098-1136(199803)22:3<272::AID-GLIA6>3.0.CO;2-7 9482213

[B27] Pierre K Pellerin L Monocarboxylate transporters in the central nervous system: distribution, regulation and function J Neurochem 2005 10 1 14 15953344 10.1111/j.1471-4159.2005.03168.x

[B28] Pellerin L Bergersen LH Halestrap AP Pierre K Cellular and subcellular distribution of monocarboxylate transporters in cultured brain cells and in the adult brain J Neurosci Res 2005 10 55 64 10.1002/jnr.20307 15573400

[B29] Cortes-Campos C Elizondo R Carril C Martinez F Boric K Nualart F Garcia-Robles MA MCT2 expression and lactate influx in anorexigenic and orexigenic neurons of the arcuate nucleus PLoS One 2013 10 e62532 10.1371/journal.pone.0062532 23638108 PMC3637215

[B30] Sun X Shih AY Johannssen HC Erb H Li P Murphy TH Two-photon imaging of glutathione levels in intact brain indicates enhanced redox buffering in developing neurons and cells at the cerebrospinal fluid and blood–brain interface J Biol Chem 2006 10 17420 17431 10.1074/jbc.M601567200 16624809

[B31] Veening JG Barendregt HP The regulation of brain states by neuroactive substances distributed via the cerebrospinal fluid; a review Cerebrospinal Fluid Res 2010 10 1 10.1186/1743-8454-7-1 20157443 PMC2821375

